# What interval of daily pain assessment is required to reliably diagnose chronic pain in SCD? The Pain in Sickle Cell Epidemiology Study

**DOI:** 10.1093/jscdis/yoae011

**Published:** 2024-10-23

**Authors:** Wally Renee Smith, Donna K McClish, Cecelia Valrie, India Sisler

**Affiliations:** Division of General Internal Medicine, Virginia Commonwealth University, Richmond, VA 23219, United States; Departments of Medicine and Biostatistics, Virginia Commonwealth University, Richmond, VA 23284, United States; Department of Psychology, Virginia Commonwealth University, Richmond, VA 23284, United States; Department of Internal Medicine, Virginia Commonwealth University, Richmond, VA 23284, United States; VCU iCubed Culture, Race, and Health Core, Office of Institutional Equity, Effectiveness and Success, Richmond, VA 23284, United States; Division of Pediatric Hematology/Oncology and Stem Cell Transplant, Children’s Hospital of Richmond at VCU, Richmond, VA 23219, United States

**Keywords:** SCD, epidemiology, chronic pain, diaries, ecological momentary assessment, test performance

## Abstract

**Objectives:**

Chronic pain in SCD has been defined as pain on most days over 6 months. In the landmark Pain in Sickle Cell Epidemiology Study, 60% of patients submitted <5 of the expected 6 months of pain diaries. Identifying chronic SCD pain using this long daily assessment interval is impractical. We therefore examined whether shorter, less burdensome intervals could accurately identify chronic SCD pain.

**Methods:**

As the gold-standard sample, we chose the 116 Pain in Sickle Cell Epidemiology Study patients who submitted >5 months of diaries (153) and >49% of diaries during all months from 1 to 4. Using the same dataset, we tested daily diary assessment over shorter intervals: 2 weeks, 1 month, 2 months, 3 months, and 4 months. We defined chronic pain as intensity rated as >0 on >50% of diary days, regardless of interval. We then calculated the sensitivity and specificity of each diary interval.

**Results:**

Among the gold-standard sample, 51.3% of patients had diary-defined chronic pain. Collection intervals of 2 months or more yielded similar chronic pain prevalences with identically high sensitivity (98.3%) and specificity (93%). Intervals of 1 month and 2 weeks yielded increasingly lower specificity (80.7%, 73.7%, respectively), but preserved sensitivity (≥96.6%).

**Conclusion:**

In the Pain in Sickle Cell Epidemiology Study, intervals of 2 months or more of daily diary collection yielded high sensitivity and specificity, compared to an interval of 5-6 months. One may reasonably diagnose chronic SCD pain using 2 months of daily diaries.

## Lay Summary

Chronic pain is prevalent in SCD. The gold-standard definition of chronic pain in SCD is pain on most days over 6 months. But completing diaries over 6 months can be burdensome and lengthy. Investigators therefore analyzed how few months of daily diaries were necessary to make the diagnosis of chronic pain accurately. We found that with just 2 months of data, we were able to correctly identify people with and without chronic pain 93%-98% of the time as compared to when we had 5-6 months of data. Diagnosing chronic pain in SCD will thus become more practical and may therefore be done more often using this easier, shorter method.

## INTRODUCTION

SCD is an extraordinarily painful, vaso-occlusive, genetic hemoglobinopathy affecting those of African, Mediterranean, and Asian descent.^1^^,^^2^ The hallmark clinical presentations are painful, disabling *vaso-occlusive crises (VOCs)* that may lead to Emergency Department use or hospitalization,^3^ correlate with mortality,^4^ and can be reduced with hydroxyurea,^5^ crizanlizumab,^6^ pharmaceutical-grade L-glutamine,^7^^,^^8^ and hematopoietic stem cell transplantation^9^ or gene therapy.^10^^,^^11^

Besides acute pain largely from VOCs, for which there is now a research consensus definition from the ACTTION-APS Pain Taxonomy (AAPT),^12^ SCD patients may also suffer from chronic pain, defined by the AAPT as pain on most days over the past 6 months, along with evidence of at least 1 sign of pain sensitivity (eg, palpation causes pain or tenderness, movement causes pain, or decreased range of motion).^13^^,^^14^ These AAPT definitions do not require the use of daily pain diaries but were based on analyses of data from the landmark Pain in Sickle Cell Epidemiology Study (PiSCES), which used daily pain diaries.^15^ In the PiSCES study, over half of SCD adults reported chronic pain using daily diaries, and 30% had pain essentially daily.^16^

Distinguishing whether chronic SCD pain is present, with or without acute SCD pain, is important. First, phenotyping chronic vs acute vs acute-on-chronic SCD pain has major treatment implications.^17–19^ Treatment for acute SCD pain, specifically VOCs, differs significantly from treatment for chronic SCD pain.^20–27^ Second, chronic SCD pain may be just as morbid as acute pain,^28–36^ though it is less recognized as morbid and is underdiagnosed and underemphasized. For example, disability determination for patients with SCD is still based on demonstration of severe anemia or a large number of VOCs or hospitalizations for VOC pain. Chronic pain is not mentioned.^37^

Third, patients with chronic pain vs only acute pain might respond differently to various SCD therapies, including transplantation and gene therapy. In one study, 83% of patients with chronic SCD pain of mixed subtypes still were on opioids after hematopoietic stem cell transplantation.^38^ It thus may be important to qualify patients for clinical trials, or subcategorize patients already enrolled in trials, according to whether they have chronic SCD pain.

Even though the above research definitions of acute and chronic SCD pain are published, the ideal measurement period and ideal methods to define acute and chronic pain in persons living with SCD have not yet been fully validated. In order to appeal most to investigators, clinicians, patients, employers, and policy-makers, the measurement period for either acute or chronic SCD pain should afford the lowest possible respondent burden and shortest possible interval of assessment, while still maintaining strong validity. And the measurement method should be the most valid and least biased. Telescoping, fabricating, and forgetting are each prevalent, and may contribute to recall bias, in the medical history.^39^ A post-hoc single historical recall of pain experienced over a long interval is associated with telescoping and inaccurate pain recall for conditions like temporomandibular joint syndrome^40^ and facial pain.^41^ This may extend to SCD, and may explain why the most prevalent SCD pain measures are standardized daily pain diaries.^35^^,^^42–53^ Diaries are a form of Ecological Momentary Assessment,^54^ considered to be less prone to telescoping, fabricating, and forgetting. Diaries can be used to measure outcomes of SCD therapy, for example in the Multicenter Study of Hydroxyurea,^55^ a trial of senicapoc,^56^ and cohort studies.^57^^,^^58^^,^^59^ Paper and pencil SCD pain diaries^60–62^ have largely been supplanted by electronic diaries^63–66^ and sometimes paired with actigraphy.^67–69^

But operationalizing the current 5-6 month gold-standard definition of chronic SCD pain by collecting daily diaries imposes an overbearing respondent burden. Indeed, more than half of PiSCES participants could not be assessed for chronic pain using the gold-standard definition.^16^ Similarly, the length of enrollment for some SCD clinical trials is often 6 months or less, so determining trial eligibility based on chronic SCD pain would take as long as the trial itself. Even using daily diaries to operationalize a 3-month interval, the interval for defining chronic pain suggested by the International Association for the Study of Pain (IASP)^70^ may be impractical for most. A shorter daily diary assessment interval upon which to reliably base a definition of chronic SCD pain would reduce respondent burden for research studies and would improve the frequency and accuracy of pain phenotyping for all purposes.

We therefore hypothesized that giving adults living with SCD daily diaries for periods much shorter than 6 months would help determine the presence of a chronic pain phenotype, with similar validity to 6 months of diary collection. To explore this, we examined various intervals of diary assessment in order to determine how long patients must complete daily diaries to diagnose whether they have chronic SCD pain.

## MATERIALS AND METHODS

The methods of PiSCES have been described in detail elsewhere.^15^^,^^16^^,^^71^ PiSCES was an epidemiological study of the relationship among various measures of pain and utilization in SCD. We enrolled 307 patients from July 2002 through August 2004. We collected baseline information (including demographic characteristics and medical history), laboratory data (blood and urine samples), and encouraged patients to complete daily pain diaries for up to 6 months. We recruited patients 16 years of age or older from across Virginia, mostly from the Richmond and Tidewater areas. Both the study and our recruitment methods were approved by the Institutional Review Board of Virginia Commonwealth University, Richmond, Virginia, and patients gave informed consent. Patients received routine care for their SCD from either community-based physicians or sickle cell specialist physicians associated with academic medical centers (2 physicians at Virginia Commonwealth University serving the Richmond area and 1 physician associated with Eastern Virginia Medical School, Norfolk, Virginia, serving the Tidewater region). Emergent care for the cohort was provided in the emergency departments regardless of the patients’ usual source of ambulatory care. No day hospitals for SCD were located in the region.

### Diary data

Patients completed paper and pencil daily diaries for up to 6 months (188 days). They were encouraged (at the initial baseline visit and with weekly reminder calls by study staff for each week they were enrolled) to complete the diary each day and return it by mail using provided stamped envelopes. They received payment for each returned diary, with a higher payment in the latter 2 months of the study to encourage study completion. Among other things, we asked patients to report in the previous 24 hours their worst sickle cell pain intensity on a 10-point Likert numeric rating scale (NRS) anchored from 0 (none) to 9 (unbearable). We used this scale, rather than the typical 11-point, 0-10 NRS,^72^ so that the PiSCES study results could be compared with results of the Multicenter Study of Hydroxyurea.^55^ Also, on each daily diary, patients could endorse (using a check box) the item, “yesterday, I was in a crisis.” VOCs were thus self-defined by each patient. Further, patients endorsed whether they had taken medication for their pain (and if so, what), had gone for an unscheduled physician visit or emergency department visit, or had been hospitalized because of sickle cell pain.

### Measurements and statistical analysis

#### Assessment strategies

Subjects in PiSCES were variably adherent with completing daily diaries. For this analysis, we chose a sample of individuals who had submitted at least 153 diaries with non-missing values marked for pain (equivalent to 5-6 months’ worth of daily diary data). To ensure the best gold-standard sample, that is, the most accurate and complete sample of daily pain measures obtainable, subjects were also excluded if they were missing at least 50% of diaries in any of months 1-4, even if they had a sufficient number of diaries overall.

For purposes of analysis, we defined a pain day as any day on which patients rated pain intensity as greater than 0. We defined a crisis day as any day when patients endorsed the crisis item on a diary and a utilization day if the subject indicated on the diary that they had gone to the emergency department or had been admitted to the hospital. We counted the number of pain days, crisis days, and utilization days during the study period, and this total served as the numerator for calculation of the percentage of pain, crisis, and utilization days. We calculated the mean pain intensity (0-9 Likert NRS) over all days, using the total of returned diaries as the denominator. We also calculated the mean pain intensity on all pain days. Note that when there were no pain days for a given patient, we could make no estimate of mean pain on pain days only.

We examined daily diary assessment over several subsets of less burdensome, shorter intervals, always beginning on day 1 of assessment, to mimic what would be done clinically. We chose the time frames of 2 weeks (2w), 1 month (1m), 2 months (2m), 3 months (3m), and 4 months (4m).

To operationalize the definition of chronic pain, we used a definition of pain severity rated as >0 on >50% of diary days, regardless of interval. To determine if using diaries collected over a shorter period of time would still classify patients correctly, we calculated the sensitivity and the number of false negatives, as well as the specificity and number of false positives, of each tested diary interval.

#### Statistical analysis

Descriptive statistics were presented as frequency (percent) or median and interquartile range (IQR), the latter due to skewness of the continuous variables. No statistical comparisons were made between those with and without chronic pain in the gold-standard sample, as that was not relevant to the thesis of this study. Potential selection bias of the gold-standard sample was first assessed by comparing characteristics of the gold-standard sample with the remainder of the 307 subjects enrolled in the PiSCES study. Second, since some of these subjects did not submit diaries, and others submitted few diaries, we also compared the gold-standard sample to the well-characterized PiSCES sample of *n* = 232 subjects who submitted at least 30 diaries.^16^ The gold-standard sample was compared to other subjects using either Chi-square test for categorical variables or Wilcoxon rank sum test for continuous variables (age as well as pain-related variables). We also constructed a histogram of the number of diaries completed by all subjects enrolled in PiSCES to examine the distribution of diary completions.

## RESULTS

**Figure 1.**
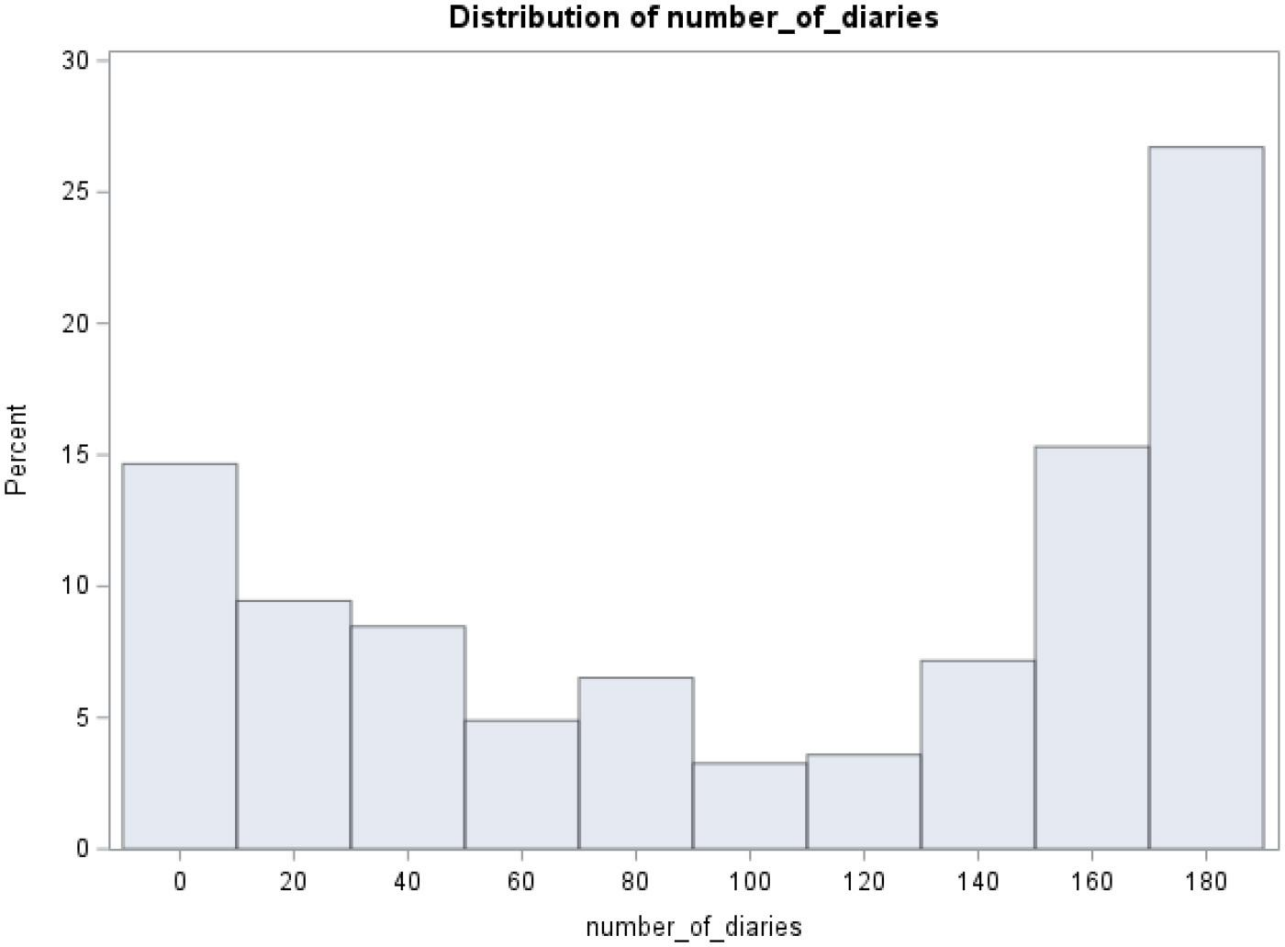
Distribution of the number of diaries completed by the 307 subjects enrolled in PiSCES. Some patients submitted no diaries. Each bar represents an interval of 20 diary days.

There were 307 unique individuals who completed a baseline survey in the PiSCES study. Figure 1 is a histogram of the number of diaries completed by all 307 subjects. Twenty-five subjects never submitted a pain rating in a diary; 18.9% submitted 2 weeks or less of diaries; 24.8% submitted 1 month or less; 36.2% submitted 2 months or less; 44.3% submitted 3 months or less; 49.5% submitted 4 months or less, and; 59.2% submitted <5 months of diaries. Of the 282 who submitted pain diaries, 124 indicated whether or not they had pain on at least 5 months of diaries. However, 8 of these subjects were further excluded due to not completing over half their diaries in 1 or more of the first 4 months, resulting in 116 subjects constituting the gold-standard sample. Of these, 59 reported pain on >50% of days and were thus classified as having chronic pain. Table 1 describes the gold-standard sample by whether they were classified as chronic pain. The median age of the gold-standard sample was 36. Gold-standard subjects were most often female (60.3%), single or never married (56.9%), and of SS or S beta thalassemia genotype (73.3%). A slim majority had either gone to college (37.9%) or graduated college (13.8%). The median number of diaries completed was 177. The mean pain intensity was 1.7 (0-9 scale) overall and was 4.3 on pain days. Pain was reported on 52.7% of days, crises were reported on 4.3% of days, and visits to the ED or hospital admission were reported on a median of 1.1% of diary days.

**Table 1.** Description of gold-standard sample—overall, and by presence or absence of chronic pain.

Characteristic	Full sample, *N* = 116	Chronic pain, *N* = 59	No chronic pain *N* = 57
Demographics			
Age, median (IQR)	36.0 (16.5)	39.0 (10.0)	30.0 (21.0)
Gender			
Female	70 (60.3)	37 (62.7)	33 (57.9)
Male	46 (39.7)	22 (37.3)	24 (42.1)
Marital status			
Married	33 (28.5)	22 (37.3)	11 (19.3)
Single	66 (56.9)	28 (47.5)	38 (66.7)
Divorced/separated/widow	17 (14.7)	9 (15.2)	8 (14.0)
Education			
<HS	17 (14.7)	7 (11.9)	10 (17.5)
HS	39 (33.6)	15 (25.4)	24 (42.1)
Some college	44 (37.9)	29 (49.1)	15 (26.3)
College grad	16 (13.8)	8 (13.6)	8 (14.0)
Income			
<10 000	39 (34.2)	22 (37.3)	17 (30.9)
10 000-19 999	31 (27.2)	20 (33.9)	11 (20.0)
20 000-29 000	17 (14.9)	9 (15.2)	8 (14.5)
≥30 000	27 (23.7)	8 (13.6)	19 (34.5)
Clinical			
Genotype			
SS/B-thal	85 (73.3)	47 (79.7)	38 (66.7)
SC/SB+thal	31 (26.7)	12 (20.3)	19 (33.3)
Pain related, median (IQR)			
#diary days	177 (14)	177 (15.0)	179 (14.0)
Mean pain—all days	1.7 (3.7)	4.0 (2.1)	0.3 (0.8)
Mean pain—pain days	4.3 (2.0)	4.7 (1.7)	3.5 (1.6)
% pain days	52.7 (89.1)	97.1 (21.0)	8.2 (21.0)
% crisis days	4.3 (14.2)	9.5 (34.9)	1.1 (5.9)
% days ED/hospital admits	1.1 (4.1)	2.9 (6.7)	0.0 (1.1)

Presented as frequency (%) of median (IQR: interquartile range) for continuous variables.

Within the gold-standard sample, 50.9% of patients were classified as chronic pain based on all diaries submitted. Table 2 shows, by tested diary interval, the variability in: the percent of patients diagnosed with chronic pain; the sensitivity as well as the number of false negatives; and the specificity as well as the number of false positives. Intervals of 2 months or more of daily diaries yielded chronic pain percentage estimates with both high sensitivity (98.3%) and high specificity (93%), compared to the gold-standard sample. Tested intervals of 1 month and 2 weeks yielded increasingly lower specificity, that is, increasingly more false-positive indications of chronic pain, as the interval shortened. In order to completely consider intervals shorter than 2 months, which provided good specificity, we also checked a 6-week interval, but specificity was still not as high as found when collecting diaries for at least 2 months (specificity was 87.7% with 7 false positives). Note that each interval still yielded high sensitivity—few patients with chronic pain in the gold-standard sample were missed, even at 2 weeks. Results were very similar even if data from all 124 participants with 5 or more months of diaries were used.

**Table 2.** Sensitivity (number of false negatives) and specificity (number of false positives) using various diary intervals for determining the presence of chronic SCD pain, the latter defined as pain on more than 50% of days over 6 months.

Time frame	Percent patients with chronic pain	Sensitivity (# false positives)	Specificity (# false negatives)
2 weeks	62.1	96.6 (2)	73.7 (15)
1 month	59.5	98.3 (1)	80.7 (11)
2 months	53.4	98.3 (1)	93.0 (4)
3 months	53.4	98.3 (1)	93.0 (4)
4 months	53.4	98.3 (1)	93.0 (4)

*N* = 116, a subset of the Pain in Sickle Cell Epidemiology study (PiSCES). Two months or more diaries yields both good sensitivity and specificity. Gold-standard % of patients with chronic pain is 50.9.

### Potential bias of the gold-standard sample

We compared the gold-standard sample of 116 with the remaining 191 subjects enrolled in PiSCES to test for selection bias. There were no significant differences in demographics or clinical characteristics between the 2 samples. The median number of diaries submitted by the 191 excluded subjects was 44. Based on the *n* = 166 subjects of the 191 not in the gold-standard sample who submitted diaries (median of 55.5 days), the only significant differences were that the gold-standard subjects had lower mean pain (median [IQR]: 1.7 [3.8] vs 2.4 [4.2], *P* = .0385) and less frequent crisis days (median [IQR]: 4.3% [14.2] vs 8.2% [44.5], *P* = .0256). We also compared the 116 gold-standard sample with the remaining 116 subjects of the PiSCES sample who submitted at least 30 diaries and were included in the primary PiSCES results publication (total *n* = 232).^44^ The only significant difference between the 2 samples was that patients in the gold-standard sample submitted more diaries, and their mean pain was marginally significantly lower (*P* = .06). There was no significant difference in the percentage (prevalence) of crisis days (see Tables S1 and S2).

## DISCUSSION

We found that, for determining the presence of chronic SCD pain, defined as pain on more than 50% of days enrolled in PiSCES, tested intervals of 2 months or more of paper daily diary collection yield both very good sensitivity and very good specificity, compared to intervals of 5-6 months. Tested intervals of 1 month and 2 weeks yielded increasingly lower specificity, that is, increasingly more false-positive indications of chronic pain as the interval shortened. However, each interval still yielded high sensitivity—few patients with gold-standard chronic pain were missed, even at 2 weeks.

Our results should bring comfort to investigators and clinicians struggling to collect SCD daily pain diaries for extended periods. Sixty-four percent of the original PiSCES sample of 307 submitted at least 2 months (60 days) of diaries, vs only 40.7% who submitted at least 5 months of diaries. We showed in an earlier PiSCES publication that, using pain intensity measures that summarized all diaries as the gold-standard sample, one can select from among various intermittent sampling strategies to get a summary measure of pain intensity.^73^ These results show one can also phenotype SCD pain as chronic using a briefer sampling strategy.

Limitations of our study include undetected bias of the sample we deemed as the gold-standard sample—only individuals who faithfully completed nearly all required diaries. This adherent sample might not be representative of people in general living with SCD. Also, our entire population was drawn from patients attending sickle cell specialty centers in academic medical centers, who may be more ill, may be hospitalized or seen more in clinic, or may otherwise not represent adults in general living with SCD. A further limitation is that the diaries for this study were done on paper, which would allow information to be completed retrospectively. Newer diary information collection methods focus on electronic means (generally mobile devices), which may include prompts to improve adherence as well as to eliminate biases associated with retrospective reporting. Yet adherence rates with electronic diaries in recent studies have not shown notable improvement over rates seen with paper diaries.^67^

To confirm that most investigators are opting not to collect 3-6 months of SCD pain diaries, as well as the struggle to get daily diary adherence for 6 months, we surveyed the length or interval of diary assessment used recently in studies of SCD. Palermo et al. conducted a trial of cognitive behavioral therapy for SCD youth and adolescents.^26^ The intervention period was 12 weeks, but outcomes were assessed electronically at baseline, immediately after completion of the intervention at 12 weeks, and at a 6-month post intervention assessment. At each of these assessments, youth and adolescents completed electronic daily pain diaries for only 7 days, and these diaries used an 11-point NRS to assess intensity. Like Palermo, most diary studies in SCD we reviewed used no more than 14 continuous days of collection.^74–76^ Exceptions were notable. Valrie et al. asked for electronic diaries for 4 weeks from youth with SCD. Fully 72% completed at least 2 weeks of diaries, and of those with 2 weeks of diaries, their average adherence rate was 81.9% of diary days.^39^ Bediako and Wang asked for electronic diaries for up to 42 consecutive days. They did not report adherence with daily diaries.^77^ Reinman et al.^78^ and Johnston et al.,^79^ in 2 publications on the same study, asked for electronic diaries for 8 consecutive weeks, and achieved a 58.5% adherence rate to diary completion. McGill et al.^80^ asked for twice-daily electronic diaries of pain severity or interference over 3 months, and achieved completion of >7 days of daily diaries and at least 25% of daily pain diaries in 77/85 eligible patients. Idris et al.^81^ asked Nigerian men with SCD to complete a paper priapism event diary for at least 3 months, but noticed signs of decline in completing the diary at 3 months. Bakshi et al.^82^ developed an on-line electronic diary especially for SCD patients, but had poor success in getting adherence in a study of yoga for SCD, so that only 1 patient attempted to use the diary. The length of data collection was to be 7 days.^83^

Pittman et al. conducting the ELLIPSIS study asked for electronic diaries for 6 months, but only 10/37 enrolled patients completed their diary more than 80% of the time. The average diary completion rate for completers was 88.6%, but for non‐completers was 57.4%.^84^^,^^85^

Based on our analysis, we believe that studies that used daily diaries for approximately 14 days would have yielded an expected sensitivity of 96.6% and therefore would have been useful to screen and include patients with chronic pain. On the other hand, those same studies would not have been as useful to exclude patients with chronic pain, since their expected specificity was only 73.7%.

Prior to performing these analyses, we were aware that many clinical trials of disease modifiers for SCD used a ceiling number of annual VOCs as an exclusion criterion or used a minimum number of annual VOCs as an inclusion criterion.^5^^,^^6^^,^^8^ This thus raised the prospect of substituting, instead of a threshold of pain days, an annualized threshold of the percent or number of crisis days, the number of crisis episodes, the number of utilization days, or the number of utilization episodes as proxy definitions of chronic pain. The frequency of these alternative measures undoubtedly correlated with the presence of chronic pain as measured by pain day frequency and with the frequency of VOCs. Testing the performance characteristics of these measures in shorter intervals of pain diaries was attempted but abandoned as part of the current study. These events were absent or rare during short intervals, making testing extremely prone to sampling bias. Future work could consider these alternate definitions.

Our results are important because of their implications for treatment, research, and clinical phenotyping. The PhenX toolkit (https://www.phenxtoolkit.org/collections/scd) lists and endorses several survey batteries for assessing and phenotyping SCD pain. A major phenotypic divide in the PhenX taxonomy and prior taxonomies for pain classification in SCD is that between acute SCD pain^41^ and chronic SCD pain,^42^ which are treated differently. For the former, guidelines advocate early and intense use of opioids with the goal of shortening hospital stays and rapidly decreasing pain intensity^50^ For the latter, coping and adjustment are often the focus, rather than eradication.^86^ It is important to distinguish between these in individual patients, who require individualized pain plans^87^ to manage their pain. Some patients with chronic pain, as well as some with acute pain, have associated psychological and social complications, which become clinically nearly indistinguishable from their pain. We and others have described how SCD pain is intertwined with depression catastrophizing,^88–90^ somatizing^91^, comorbid depression and anxiety,^32^ alcoholism,^92^ kinesiophobia,^93^ disturbed sleep or sleep-disordered breathing,^69^^,^^94^^,^^95^ and of course opioid tolerance, misuse, and central sensitization.^65^^,^^96^^,^^97^ Assessing some or all of these may help clinicians choose multimodal plans of attack as part of an individualized pain plan.

## CONCLUSION

We conclude that clinicians and researchers may reasonably choose to adopt 2 months of diary collection, as a matter of practicality, to reduce respondent burden when phenotyping SCD pain as acute vs chronic. Future study should determine required sampling intervals for other pain phenotyping tasks such as pain function, pain quality, and pain location.

## Supplementary Material

yoae011_Supplementary_Data

## Data Availability

Data used for these analyses are archived at a VCU file storage facility and are freely available upon request to the first (corresponding author). Please allow 2-4 weeks for processing time.

## References

[1] Serjeant GR. One hundred years of sickle cell disease. Br J Haematol. 2010;151(5):425-429. 10.1111/j.1365-2141.2010.08419.x 20955412

[2] Steinberg MH. Management of sickle cell disease. N Engl J Med. 1999;340(13):1021-1030. 10099145 10.1056/NEJM199904013401307

[3] Aisiku IP, Smith WR, McClish DK, et al Comparisons of high versus low emergency department utilizers in sickle cell disease. Ann Emerg Med. 2009;53(5):587-593. 18926599 10.1016/j.annemergmed.2008.07.050

[4] Platt OS, Thorington BD, Brambilla DJ, et al Pain in SCD. Rates and risk factors. N Engl J Med. 1991;325(1):11-16. 1710777 10.1056/NEJM199107043250103

[5] Charache S, Terrin ML, Moore RD, et al Effect of hydroxyurea on frequency of painful crises in sickle cell anemia. Investigators of the Multicenter Study of Hydroxyurea in Sickle Cell Anemia. N Engl J Med. 1995;332(20):1317-1322. 7715639 10.1056/NEJM199505183322001

[6] Ataga KI, Kutlar A, Kanter J, et al Crizanlizumab for the prevention of pain crises in sickle cell disease. N Engl J Med. 2017;376(5):429-439. 10.1056/NEJMoa1611770 27959701 PMC5481200

[7] Food and Drug Administration. FDA approved L-glutamine powder for the treatment of sickle cell disease. Accessed November 19, 2024. https://www.fda.gov/Drugs/InformationOnDrugs/ApprovedDrugs/ucm566097.htm

[8] Clinical Trials.gov. A phase III, prospective, randomized, double-blind, placebo-controlled, parallel-group, multicenter study of L glutamine therapy for sickle cell anemia and sickle ß0-thalassemia. NCT01179217. Accessed January 24, 2010. http://clinicaltrials.gov.

[9] Krishnamurti L, Neuberg DS, Sullivan KM, et al Bone marrow transplantation for adolescents and young adults with sickle cell disease: results of a prospective multicenter pilot study. Am J Hematol. 2019;94(4):446-454. 10.1002/ajh.25401 30637784 PMC6542639

[10] Kanter J, Thompson AA, Pierciey FJ Jr, et al Lovo-cel gene therapy for sickle cell disease: treatment process evolution and outcomes in the initial groups of the HGB-206 study. Am J Hematol. 2023;98(1):11-22. 10.1002/ajh.26741 36161320 PMC10092845

[11] Dimitrievska M, Bansal D, Vitale M, et al Revolutionising healing: gene editing’s breakthrough against sickle cell disease. Blood Rev. 2024;65:101185. 10.1016/j.blre.2024.101185 38493007

[12] Field JJ, Ballas SK, Campbell CM, et al AAAPT diagnostic criteria for acute sickle cell disease pain. J Pain. 2019;20(7):746-759. 30578848 10.1016/j.jpain.2018.12.003

[13] Dampier C, Palermo TM, Darbari DS, Hassell K, Smith W, Zempsky W. AAPT diagnostic criteria for chronic sickle cell disease pain. J Pain. 2017;18(5):490-498. 28065813 10.1016/j.jpain.2016.12.016

[14] Smith WR, Scherer M. Sickle-cell pain: advances in epidemiology and etiology. Hematology Am Soc Hematol Educ Program. 2010;2010:409-415. 10.1182/asheducation-2010.1.409 21239827

[15] Smith WR, Bovbjerg VE, Penberthy LT, et al Understanding pain and improving management of sickle cell disease: the PiSCES study. J Natl Med Assoc. 2005;97(2):183-193. 15712781 PMC2568749

[16] Smith WR, McClish DK, Penberthy LT, et al Daily assessment of pain in adults with sickle cell disease. Ann Intern Med. 2008;148(2):94-101. 18195334 10.7326/0003-4819-148-2-200801150-00004

[17] Kenney MO, Smith WR. Moving toward a multimodal analgesic regimen for acute sickle cell pain with non-opioid analgesic adjuncts: a narrative review. J Pain Res. 2022;15:879-894. 10.2147/JPR.S343069 35386424 PMC8979590

[18] Smith WR, Valrie CR, Jaja C, Kenney MO. Precision, integrative medicine for pain management in sickle cell disease. Front Pain Res (Lausanne). 2023;4:1279361. 10.3389/fpain.2023.1279361 38028431 PMC10666191

[19] Kenney MO, Wilson S, Shah N, et al Biopsychosocial factors associated with pain and pain-related outcomes in adults and children with sickle cell disease: a multivariable analysis of the GRNDaD multicenter registry. J Pain. 2024;25(1):153-164. 10.1016/j.jpain.2023.07.029 37544393 PMC11261903

[20] Yawn BP, Buchanan GR, Afenyi-Annan AN, et al Management of sickle cell disease: summary of the 2014 evidence-based report by expert panel members. JAMA. 2014;312(10):1033-1048. 10.1001/jama.2014.10517; Erratum in: JAMA. 2014;312(18):1932; Erratum in: JAMA. 2015;313(7):729. 25203083

[21] Brandow AM, Carroll CP, Creary S, et al American Society of Hematology 2020 guidelines for sickle cell disease: management of acute and chronic pain. Blood Adv. 2020;4(12):2656-2701. 10.1182/bloodadvances.2020001851 PMC7322963 32559294

[22] Evensen CT, Treadwell MJ, Keller S, et al Quality of care in sickle cell disease: cross-sectional study and development of a measure for adults reporting on ambulatory and emergency department care. Medicine (Baltimore). 2016;95(35):e4528. 10.1097/MD.0000000000004528 27583862 PMC5008546

[23] Tanabe P, Bosworth HB, Crawford RD, et al Time to pain relief: a randomized controlled trial in the emergency department during vaso-occlusive episodes in sickle cell disease. Eur J Haematol. 2023;110(5):518-526. 10.1111/ejh.13924 36602417 PMC10073280

[24] Kanjee Z, Achebe MO, Smith WR, Burns RB. How would you treat this patient with acute and chronic pain from sickle cell disease?: grand rounds discussion from Beth Israel Deaconess Medical Center. Ann Intern Med. 2022;175(4):566-573. 10.7326/M22-0038 35404671

[25] McDonagh MS, Selph SS, Buckley DI, et al Nonopioid Pharmacologic Treatments for Chronic Pain [Internet]. Report No.: 20-EHC010. Agency for Healthcare Research and Quality (US); 2020. 32338847

[26] Palermo TM, Lalloo C, Zhou C, et al A cognitive-behavioral digital health intervention for sickle cell disease pain in adolescents: a randomized, controlled, multicenter trial. Pain. 2024;165(1):164-176. 10.1097/j.pain.0000000000003009 37733479 PMC10723646

[27] Gil KM, Wilson JJ, Edens JL, et al Effects of cognitive coping skills training on coping strategies and experimental pain sensitivity in African American adults with sickle cell disease. Health Psychol. 1996;15(1):3-10. 10.1037//0278-6133.15.1.3 8788535

[28] Mucalo L, Field JJ, Highland J, et al Preliminary construct validity of patient-reported outcomes to assess chronic pain in adults with sickle cell disease. Blood Adv. 2023;7(14):3658-3665. 37058480 10.1182/bloodadvances.2023009707 PMC10365933

[29] Von Korff M, DeBar LL, Krebs EE, Kerns RD, Deyo RA, Keefe FJ. Graded chronic pain scale revised: mild, bothersome, and high-impact chronic pain. Pain. 2020;161(3):651-661. 10.1097/j.pain.0000000000001758 31764390 PMC7097879

[30] Jagtiani A, Chou E, Gillespie SE, et al High-impact chronic pain in sickle cell disease: insights from the Pain in Sickle Cell Epidemiology Study (PiSCES). PAIN. 2024;165(10):2364-2369. 38787626 10.1097/j.pain.0000000000003262 PMC11404329

[31] Platt OS, Brambilla DJ, Rosse WF, et al Mortality in sickle cell disease. Life expectancy and risk factors for early death. N Engl J Med. 1994;330(23):1639-1644. 10.1056/NEJM199406093302303 7993409

[32] Levenson JL, McClish DK, Dahman BA, et al Depression and anxiety in adults with sickle cell disease: the PiSCES project. Psychosom Med. 2008;70(2):192-196. 10.1097/PSY.0b013e31815ff5c5 18158366

[33] Edwards CL, Green M, Wellington CC, et al Depression, suicidal ideation, and attempts in Black patients with sickle cell disease. J Natl Med Assoc. 2009;101(11):1090-1095. 10.1016/S0027-9684(15)31103-2 19998636

[34] Swirsky ES, Boyd AD, Gu C, et al Monitoring and responding to signals of suicidal ideation in pragmatic clinical trials: lessons from the GRACE trial for Chronic Sickle Cell Disease Pain. Contemp Clin Trials Commun. 2023;36:101218. 10.1016/j.conctc.2023.101218 37842321 PMC10569945

[35] Gil KM, Carson JW, Porter LS, Scipio C, Bediako SM, Orringer E. Daily mood and stress predict pain, health care use, and work activity in African American adults with sickle-cell disease. Health Psychol. 2004;23(3):267-274. 10.1037/0278-6133.23.3.267 15099167

[36] Holdford D, Vendetti N, Sop DM, Johnson S, Smith WR. Indirect economic burden of sickle cell disease. Value Health. 2021;24(8):1095-1101. 10.1016/j.jval.2021.02.014 34372974

[37] Disability Evaluation Under Social Security. 7.00. Hematological disorders—adult. Accessed April 9, 2024. https://www.ssa.gov/disability/professionals/bluebook/7.00-HematologicalDisorders-Adult.htm

[38] Darbari DS, Liljencrantz J, Ikechi A, et al Pain and opioid use after reversal of sickle cell disease following HLA-matched sibling haematopoietic stem cell transplant. Br J Haematol. 2019;184(4):690-693. 10.1111/bjh.15169 29527656 PMC6541482

[39] Barsky AJ. Forgetting, fabricating, and telescoping: the instability of the medical history. Arch Intern Med. 2002;162(9):981-984. 10.1001/archinte.162.9.981 11996606

[40] Salé H, Hedman L, Isberg A. Accuracy of patients’ recall of temporomandibular joint pain and dysfunction after experiencing whiplash trauma: a prospective study. J Am Dent Assoc. 2010;141(7):879-886. 10.14219/jada.archive.2010.0287 20592409

[41] Raphael KG, Marbach JJ. When did your pain start?: reliability of self-reported age of onset of facial pain. Clin J Pain. 1997;13(4):352-359. 10.1097/00002508-199712000-00014 9430817

[42] Walco GA, Dampier CD. Pain in children and adolescents with sickle cell disease: a descriptive study. J Pediatr Psychol. 1990;15(5):643-658. 2283573 10.1093/jpepsy/15.5.643

[43] Gonzalez ER, Ornato JP, Ware D, Bull DS, Evens RP. Comparison of the intramuscular analgesic activity of butorphanol and morphine in patients with sickle cell disease. Ann Emerg Med. 1988;17(8):788-791. 3394980 10.1016/s0196-0644(88)80554-7

[44] Gonzales ER, Bahal N, Hansen LA, et al Intermittent injection vs. patient controlled analgesia for sickle cell crisis pain. Arch Int Med. 1991;151:1373-1378. 2064488

[45] Shapiro BS, Dinges DF, Orne EC, et al Home management of sickle cell-related pain in children and adolescents: natural history and impact on school attendance. Pain. 1995;61(1):139-144. 7644237 10.1016/0304-3959(94)00164-A

[46] Fuggle P, Shand PA, Gill LJ, Davies SC. Pain, quality of life, and coping in sickle cell disease. Arch Dis Child. 1996;75(3):199-203. 8976657 10.1136/adc.75.3.199 PMC1511691

[47] Conner-Warren RL. Pain intensity and home pain management of children with sickle cell disease. Issues Compr Pediatr Nurs. 1996;19(3):183-195. 9119714 10.3109/01460869609026860

[48] Westerman MP, Bailey K, Freels S, Schlegel R, Williamson P. Assessment of painful episode frequency in sickle-cell disease. Am J Hematol. 1997;54(3):183-188. 9067495 10.1002/(sici)1096-8652(199703)54:3<183::aid-ajh2>3.0.co;2-s

[49] Gil K, Abrams M, Phillips G, Williams D. Sickle cell disease pain: 2. Predicting health care use and activity level at 9-month follow-up. J Consult Clin Psychol. 1992;60(2):267-273. 1592957 10.1037//0022-006x.60.2.267

[50] Porter LS, Gil KM, Sedway JA, Ready J, Workman E, Thompson RJ. Pain and stress in sickle cell disease: an analysis of daily pain records. Int J Behav Med. 1998;5(3):185-203. 16250701 10.1207/s15327558ijbm0503_1

[51] Dampier C, Ely E, Brodecki D, O’Neal P. Home management of pain in sickle cell disease: a daily diary study in children and adolescents. J Pediatr Hematol Oncol. 2002;24(8):643-647. 12439036 10.1097/00043426-200211000-00008

[52] Gil KM, Porter LS, Ready J, Workman E, Sedway J, Anthony KK. Pain in children and adolescents with sickle cell disease: an analysis of daily pain diaries. Children’s Health Care. 2000;29(4):225-241.

[53] Gil KM, Carson JW, Porter LS, et al Daily stress and mood and their association with pain, health-care use, and school activity in adolescents with sickle cell disease. J Pediatr Psychol. 2003;28(5):363-373. 12808013 10.1093/jpepsy/jsg026

[54] Moskowitz DS, Young SN. Ecological momentary assessment: what it is and why it is a method of the future in clinical psychopharmacology. J Psychiatry Neurosci. 2006;31(1):13-20. 16496031 PMC1325062

[55] Charache S, Terrin ML, Moore RD, et al Design of the multicenter study of hydroxyurea in sickle cell anemia. Investigators of the Multicenter Study of Hydroxyurea. Control Clin Trials. 1995;16(6):432-446. 8925656 10.1016/s0197-2456(95)00098-4

[56] Ataga KI, Smith WR, De Castro LM, et al; ICA-17043-05 Investigators. Efficacy and safety of the Gardos channel blocker, senicapoc (ICA-17043), in patients with sickle cell anemia. Blood. 2008;111(8):3991-3997. 18192510 10.1182/blood-2007-08-110098

[57] Dampier C, Ely EB, Brodecki D, O’Neal P. Home management characteristics of pain in sickle cell disease. Pediatr Hematol Oncol. 2004;26:785-790. 15591896

[58] Dampier C, Ely B, Brodecki D, O’Neal P. Characteristics of pain managed at home in children and adolescents with sickle cell disease by using diary self-reports. J Pain. 2002;3(6):461-470. 14622732 10.1054/jpai.2002.128064

[59] Dampier C, Setty BN, Eggleston B, Brodecki D, O’Neal P, Stuart M. Vaso-occlusion in children with sickle cell disease: clinical characteristics and biologic correlates. J Pediatr Hematol Oncol. 2004;26(12):785–790. 15591896

[60] van Tuijn CF, Sins JW, Fijnvandraat K, Biemond BJ. Daily pain in adults with sickle cell disease-a different perspective. Am J Hematol. 2017;92(2):179-186. 10.1002/ajh.24612 27880985

[61] Valrie CR, Gil KM, Redding-Lallinger R, Daeschner C. Sleep in children with sickle cell disease: an analysis of daily diaries utilizing multilevel models. J Pediatr Psychol. 2007;32(7):857-861. 17400602 10.1093/jpepsy/jsm016

[62] Valrie CR, Gil KM, Redding-Lallinger R, Daeschner C. The influence of pain and stress on sleep in children with sickle cell disease. Children’s Health Care. 2007;36(4):335-353.

[63] Bakshi N, Smith ME, Ross D, Krishnamurti L. Novel metrics in the longitudinal evaluation of pain data in sickle cell disease. Clin J Pain. 2017;33(6):517-527. 10.1097/AJP.0000000000000431 27584817

[64] Hoppe C, Jacob E, Styles L, Kuypers F, Larkin S, Vichinsky E. Simvastatin reduces vaso-occlusive pain in sickle cell anaemia: a pilot efficacy trial. Br J Haematol. 2017;177(4):620-629. 10.1111/bjh.14580 28369718 PMC5435522

[65] Carroll CP, Lanzkron S, Haywood C Jr, et al Chronic opioid therapy and central sensitization in sickle cell disease. Am J Prev Med. 2016;51(1 Suppl 1):S69-S77. 10.1016/j.amepre.2016.02.012 27320469 PMC5379857

[66] Heeney MM, Hoppe CC, Abboud MR et al; DOVE Investigators. A multinational trial of prasugrel for sickle cell vaso-occlusive events. N Engl J Med. 2016;374(7):625-635. 10.1056/NEJMoa1512021 26644172

[67] Karlson CW, Baker AM, Bromberg MH, David Elkin T, Majumdar S, Palermo TM. Daily pain, physical activity, and home fluid intake in pediatric sickle cell disease. J Pediatr Psychol. 2017;42(3):335-344. 10.1093/jpepsy/jsw061 27370016

[68] Valrie CR, Kilpatrick RL, Alston K, et al Investigating the sleep-pain relationship in youth with sickle cell utilizing mHealth technology. J Pediatr Psychol. 2019;44(3):323-332. 30649539 10.1093/jpepsy/jsy105 PMC6681631

[69] Valrie CR, Alston K, Morgan K, Kilpatrick R, Sisler I, Fuh B. Pediatric sickle cell pain-sleep relationships: the roles of positive and negative affect. Health Psychol. 2021;40(11):793-802. 34914484 10.1037/hea0001144 PMC9260698

[70] International Association for the Study of Pain (IASP). Pain IASP Taxonomy. 2015. Accessed May 12, 2022. http://www.iasp-pain.org/Education/Content.aspx?ItemNumber=1698&navItemNumber=576

[71] McClish DK, Levenson JL, Penberthy LT, et al Gender differences in pain and healthcare utilization for adult sickle cell patients: the PiSCES Project. J Womens Health (Larchmt). 2006;15(2):146-154. 16536678 10.1089/jwh.2006.15.146

[72] von Baeyer CL, Spagrud LJ, McCormick JC, Choo E, Neville K, Connelly MA. Three new datasets supporting use of the Numerical Rating Scale (NRS-11) for children’s self-reports of pain intensity. PAIN. 2009;143(3):223-227. 19359097 10.1016/j.pain.2009.03.002

[73] Smith WR, McClish DK, Levenson J, et al Predictive ability of intermittent daily sickle cell pain assessment: the PiSCES project. Pain Med. 2018;19(10):1972-1981. 10.1093/pm/pnx214 29036363 PMC6176749

[74] Krishnamurti L, Arnold SD, Haight A, et al Sickle Cell Transplantation Evaluation of Long-term and Late Effects Registry (STELLAR) to compare long-term outcomes after hematopoietic cell transplantation to those in siblings without sickle cell disease and in nontransplanted individuals with sickle cell disease: design and feasibility study. JMIR Res Protoc. 2022;11(7):e36780. 10.2196/36780 35793124 PMC9301564

[75] Ellis JD, Samiei S, Neupane S, et al Sleep disruption moderates the daily dynamics of affect and pain in sickle cell disease. J Pain. 2024;25(7):104477. 10.1016/j.jpain.2024.01.342 38242332 PMC11180574

[76] Bakshi N, Astles R, Chou E, et al Multimodal phenotyping and correlates of pain following hematopoietic cell transplant in children with sickle cell disease. Pediatr Blood Cancer. 2023;70(1):e30046. 10.1002/pbc.30046 36322607 PMC9820671

[77] Bediako SM, Wang Y. Daily loneliness affects quality of life in sickle cell disease. Int J Behav Med. 2024;31(3):393-398. 10.1007/s12529-023-10247-1 38097875

[78] Reinman L, Schatz J, Johnston J, Bills S. Fatigue, stress appraisal, and emotional functioning among youth with sickle cell disease: a daily diary study. J Pediatr Psychol. 2023;48(6):562-571. 10.1093/jpepsy/jsad019 37167536 PMC10321392

[79] Johnston JD, Reinman LC, Bills SE, Schatz JC. Sleep and fatigue among youth with sickle cell disease: a daily diary study. J Behav Med. 2023;46(3):440-450. 10.1007/s10865-022-00368-5 36334167 PMC9638215

[80] McGill LS, Hamilton KR, Letzen JE et al Depressive and insomnia symptoms sequentially mediate the association between racism-based discrimination in healthcare settings and clinical pain among adults with sickle cell disease. J Pain 2023;24(4):643–654. 10.1016/j.jpain.2022.11.004 PMC10079566 36414154

[81] Idris IM, Abba A, Galadanci JA, et al Incidence and predictors of priapism events in sickle cell anemia: a diary-based analysis. Blood Adv. 2022;6(20):5676-5683. 10.1182/bloodadvances.2022007285 35816639 PMC9582584

[82] Bakshi N, Stinson JN, Ross D, et al Development, content validity, and user review of a web-based multidimensional pain diary for adolescent and young adults with sickle cell disease. Clin J Pain. 2015;31(6):580-590. 10.1097/AJP.0000000000000195 25565585

[83] Bakshi N, Cooley A, Ross D, et al A pilot study of the acceptability, feasibility and safety of yoga for chronic pain in sickle cell disease. Complement Ther Med. 2021;59:102722. 10.1016/j.ctim.2021.102722 33892094 PMC8284565

[84] Pittman D, Hines PC, Beidler DR, et al Evaluation of longitudinal pain study in sickle cell disease (ELIPSIS) by electronic patient‐reported outcomes, actigraphy, and biomarkers. Blood. 2021;137(15):2010-2020. 10.1182/blood.2020006020 33067606 PMC8057263

[85] Coyne KS, Currie BM, Callaghan M, et al Validation of patient-reported vaso-occlusive crisis day as an endpoint in sickle cell disease studies. Eur J Haematol. 2022;109(3):226-237. 10.1111/ejh.13790 35569114 PMC9542396

[86] Gil KM, Carson JW, Sedway JA, Porter LS, Schaeffer JJ, Orringer E. Follow-up of coping skills training in adults with sickle cell disease: analysis of daily pain and coping practice diaries. Health Psychol. 2000;19(1):85-90. 10.1037//0278-6133.19.1.85 10711591

[87] Krishnamurti L, Smith-Packard B, Gupta A, Campbell M, Gunawardena S, Saladino R. Impact of individualized pain plan on the emergency management of children with sickle cell disease. Pediatr Blood Cancer. 2014;61(10):1747-1753. 24962217 10.1002/pbc.25024

[88] Citero VdA, Levenson JL, McClish DK, et al The role of catastrophizing in sickle cell disease—the PiSCES project. Pain. 2007;133(1-3):39-46. 10.1016/j.pain.2007.02.008 17408858

[89] Bakshi N, Gillespie S, McClish D, McCracken C, Smith WR, Krishnamurti L. Intraindividual pain variability and phenotypes of pain in sickle cell disease: a secondary analysis from the pain in Sickle Cell Epidemiology Study. Pain. 2022;163(6):1102-1113. 10.1097/j.pain.0000000000002479 34538841 PMC9100443

[90] Kuisell C, Ploutz-Snyder R, Williams DA, et al Adolescents and young adults with sickle cell disease: nociplastic pain and pain catastrophizing as predictors of pain interference and opioid consumption. Clin J Pain. 2023;39(7):326-333. 10.1097/AJP.0000000000001119 37083638 PMC10330104

[91] Sogutlu A, Levenson JL, McClish DK, Rosef SD, Smith WR. Somatic symptom burden in adults with sickle cell disease predicts pain, depression, anxiety, health care utilization, and quality of life: the PiSCES project. Psychosomatics. 2011;52(3):272-279. 21565599 10.1016/j.psym.2011.01.010

[92] Levenson JL, McClish DK, Dahman BA, et al Alcohol abuse in sickle cell disease: the Pisces Project. Am J Addict. 2007;16(5):383-388. 10.1080/10550490701525434 17882609

[93] Pells J, Edwards CL, McDougald CS, et al Fear of movement (kinesiophobia), pain, and psychopathology in patients with sickle cell disease. Clin J Pain. 2007;23(8):707-713. 10.1097/AJP.0b013e31814da3eb 17885350

[94] Valrie CR, Alston K, Fuh B, Redding-Lallinger R, Sisler I. Sleep moderating the relationship between pain and health care use in youth with sickle cell disease. Clin J Pain. 2020;36(2):117-123. 10.1097/AJP.0000000000000783 31789829 PMC7579672

[95] Karlson CW, Barajas KG, Seals SR, et al Longitudinal predictors of pain in pediatric sickle cell disease. J Pediatr Psychol. 2023;48(6):553-561. 10.1093/jpepsy/jsad017 37043758

[96] Smith WR, McClish DK, Roberts JD, et al Prescription opioid misuse index in sickle cell patients: a brief questionnaire to assess at-risk for opioid abuse. J Opioid Manag. 2019;15(4):323-331. 10.5055/jom.2019.0517 31637684

[97] Jonassaint CR, Parchuri E, O’Brien JA, et al Mental health, pain and likelihood of opioid misuse among adults with sickle cell disease. Br J Haematol. 2024;204(3):1029-1038. 10.1111/bjh.19243 38171495 PMC10939903

