# Supplementary material for: What interval of daily pain assessment is required to reliably diagnose chronic pain in SCD? The Pain in Sickle Cell Epidemiology Study
Source: J Sick Cell Dis. 2024 Oct 23;1(1):yoae011. doi: 10.1093/jscdis/yoae011 (PMC12039818; doi:10.1093/jscdis/yoae011)
Supplement: yoae011_Supplementary_Data [file yoae011_Supplementary_Data.docx]

**Supplementary Materials**

**What Interval of Daily Pain Assessment is Required to Reliably Diagnose Chronic Pain in Sickle Cell Disease? The PiSCES Project**

**Wally Renee Smith, MD, Donna K McClish, PhD, Cecelia Valrie, PhD, India Sisler, MD**

**Virginia Commonwealth University**

*Corresponding Author: Wally **Renee** Smith, MD, wally.smith@vcuhealth.org

*Florence Neal Cooper Smith Professor of Sickle Cell Disease,*

*Vice Chair for Research, Division of General Internal Medicine,*

*Virginia Commonwealth University, Richmond, Virginia,*

Box 980306

730 E Broad St Suite 430

Richmond, VA, USA 23219

Wally.smith@vcuhealth.org

804-828-8360

ORcid ID https://orcid.org/0000-0002-4122-5367

Donna K McClish, PhD

Professor Emeritus

Departments of Medicine and Biostatistics

Virginia Commonwealth University

Richmond, VA, USA 23284

Donna.mcclish@gmail.com

[https://orcid.org/0000-0003-0344-6377](https://urldefense.com/v3/__https:/orcid.org/0000-0003-0344-6377?lang=en__;!!JqxBPMk!hS_4nGdIcixPsIdTOWG2Iz-2_1Zd9Xe_h2QgZ8SSQnLDqyIqL3VPpZ11JlBgjrro-khkEva_o12emk3kM6M5pA$)

India Sisler, MD

Associate Professor of Pediatrics

Children’s Hospital of Richmond at VCU

Division of Pediatric Hematology/Oncology and Stem Cell Transplant

1000 E Broad Street

Richmond, VA, USA 23219

(804) 828-9605

[i](mailto:India.sisler@vcuhealth.org)ndia.sisler@vcuhealth.org

Cecelia Valrie

Associate Professor, [Health Psych PhD Program](https://urldefense.com/v3/__https:/psychology.vcu.edu/graduate/health/__;!!JqxBPMk!nW-K8OzHPCUEutPOpFdeZQLienYaS6NafSqQBx5LdkASTdPtZTmmm9MksK-0LlMoTS80DqCNJwIcMCqbRX4$) | [Department of Psychology](https://urldefense.com/v3/__https:/psychology.vcu.edu/__;!!JqxBPMk!nW-K8OzHPCUEutPOpFdeZQLienYaS6NafSqQBx5LdkASTdPtZTmmm9MksK-0LlMoTS80DqCNJwIcCuydDmM$)

Adjunct Associate Professor, [Department of Internal Medicine](https://urldefense.com/v3/__https:/intmed.vcu.edu/__;!!JqxBPMk!nW-K8OzHPCUEutPOpFdeZQLienYaS6NafSqQBx5LdkASTdPtZTmmm9MksK-0LlMoTS80DqCNJwIcVvsr1no$)

Virginia Commonwealth University

Director, [Pediatric Health and Development Lab](https://urldefense.com/v3/__https:/hd.lab.vcu.edu/__;!!JqxBPMk!nW-K8OzHPCUEutPOpFdeZQLienYaS6NafSqQBx5LdkASTdPtZTmmm9MksK-0LlMoTS80DqCNJwIcHDdkB30$)

Chair, [VCU iCubed Culture, Race, and Health Core](https://urldefense.com/v3/__https:/icubed.vcu.edu/programs/culture-race-health/__;!!JqxBPMk!nW-K8OzHPCUEutPOpFdeZQLienYaS6NafSqQBx5LdkASTdPtZTmmm9MksK-0LlMoTS80DqCNJwIc4o4Xtqw$)

808 W. Franklin St., Rm 201

Richmond, VA, USA 23284

Phone: (804) 827-1562

Fax: (804) 828-2237

Email: [cvalrie@vcu.edu](mailto:cvalrie@vcu.edu)

ORCID id  <https://orcid.org/0000-0002-7204-6812>

*Corresponding Author contact:

Wally.smith@vcuhealth.org

Box 980306

730 E Broad St Suite 430

Richmond, VA, USA 23219

804-828-8360

**Funding Statement:**

*Supported by funding from:*

1. SHIP-HU Study, 1 R18 HL 112737, National Heart, Lung, and Blood Institute, NIH
2. Pain in Sickle Cell Epidemiology Study, 1 R01 HL 64122, National Heart, Lung, and Blood Institute, NIH
3. Virginia Basic and Translational Research Program in Sickle Cell Disease. 1U54HL090516, National Heart, Lung, and Blood Institute, and;
4. Sickle Cell Disease Clinical Research Network, National Heart, Lung, and Blood Institute 1U10HL083732

**Conflict of Interest**:

1. WRS is a consultant with Pfizer, Agios, Novo-Nordisk, Fulcrum, and Novartis

**Author Contribution**

ChatGPT was not used.

Wally **Renee** Smith, MD (Funding, Conceptualization, Data collection, Supervision, Formal Analysis, writing-original draft, review and editing)

Donna K McClish PhD (Conceptualization, Data collection, Supervision, Formal Analysis, writing-original draft, review and editing)

India Sisler, MD (Writing, review and editing)

Cecelia Valrie, PhD (Writing, review and editing)

**Acknowledgements:**

None

**Data Availability**

Data used for these analyses are archived at a VCU file storage facility, and are freely avaiable upon request of the first (contact author). Please allow 2-4 weeks for processing time.

**Supplemental tables**

| Characteristic | Gold-standard sample;  N=116 | Remainder of the PiSCES sample; N=191 | p-value |
| --- | --- | --- | --- |
| DEMOGRAPHICS |  |  |  |
| Age | 36.0 (16.5) | 29 (15) | 0.1023 |
| Gender |  |  |  |
| Female | 70 (60.3) | 116 (60.7) |  |
| Male | 46 (39.7) | 75 (39.3) |  |
| Marital Status |  |  | 0.0819 |
| Married | 33 (28.5) | 35 (18.3) |  |
| Single | 66 (56.9) | 131 (68.6) |  |
| Divorced/separated/widow | 17 (14.7) | 25 (13.1) |  |
| Education |  |  | 0.6363 |
| <HS | 17 (14.7) | 24 (12.6) |  |
| HS | 39 (33.6) | 78 (41.1) |  |
| Some college | 44 (37.9) | 64 (33.7) |  |
| College grad | 16 (13.8) | 24 (12.6) |  |
| Income |  |  | 0.5611 |
| <10,000 | 39 (34.2) | 77 (41.9) |  |
| 10,000-19,999 | 31 (27.2) | 40 (21.7) |  |
| 20,000-29,000 | 17 (14.9) | 27 (14.7) |  |
| >=30,000 | 27 (23.7) | 40 (21.7) |  |
| CLINICAL |  |  |  |
| Genotype |  |  | 0.9742 |
| SS/B-thal | 85 (73.3) | 130 (73.5) |  |
| SC/SB+thal | 31 (26.7) | 47 (26.5) |  |
| PAIN RELATED (median/IQR) |  |  |  |
| # diary days | 177 (14) | 44 (108) | <0.0001 |
| Mean pain – all days | 1.7 ( 3.8) | 2.4 ( 4.2) | 0.0385 |
| Mean pain - pain days | 4.3 ( 2.0) | 4.4 ( 2.5) | 0.3009 |
| % pain days | 52.7 (89.1) | 67.8 (75.3) | 0.1028 |
| % crisis days | 4.3 (14.2) | 8.2 (44.5) | 0.0256 |
| % days ED/hospital admits | 1.1 ( 4.1) | 0.0 ( 3.0) | 0.2269 |

Supplemental Table 1 Sample description comparing the gold-standard sample with the remainder of the 307 subjects enrolled in PiSCES. Presented as frequency (%) of median (IQR:Interquartile range) for continuous variables. Note that number of diary days includes those who did not submit diaries (# diary days=0); The remainder of the pain related measures are based on the n=166 of 191 who submitted at least 1 diary.

| Characteristic | Gold-standard sample  N=116 | Remainder who submitted≥30 diaries  N=116* | p-value |
| --- | --- | --- | --- |
| DEMOGRAPHICS |  |  |  |
| Age | 36.0 (16.5) | 32.5 (15.5) | 0.6250 |
| Gender |  |  | 0.6854 |
| Female | 70 (60.3) | 73 (62.9) |  |
| Male | 46 (39.7) | 43 (37.1) |  |
| Marital Status |  |  | 0.1989 |
| Married | 33 (28.5) | 22 (19.0) |  |
| Single | 66 (56.9) | 78 (67.2) |  |
| Divorced/separated/widow | 17 (14.7) | 16 (13.8) |  |
| Education |  |  | 0.3949 |
| <HS | 17 (14.7) | 11 (9.5) |  |
| HS | 39 (33.6) | 49 (42.2) |  |
| Some college | 44 (37.9) | 38 (32.8) |  |
| College grad | 16 (13.8) | 18 (15.5) |  |
| Income |  |  | 0.3804 |
| <10,000 | 39 (34.2) | 49 (43.4) |  |
| 10,000-19,999 | 31 (27.2) | 21 (18.6) |  |
| 20,000-29,000 | 17 (14.9) | 17 (15.0) |  |
| >=30,000 | 27 (23.7) | 26 (23.0) |  |
| CLINICAL |  |  |  |
| Genotype |  |  | 0.9682 |
| SS/B-thal | 85 (73.3) | 84 (73.0) |  |
| SC/SB+thal | 31 (26.7) | 31 (27.0) |  |
| PAIN RELATED (median/IQR) |  |  |  |
| # diary days | 177 (14) | 88 (82.5) | <0.0001 |
| Mean pain – all days | 1.7 ( 3.8) | 2.4 ( 4.2) | 0.0600 |
| Mean pain - pain days | 4.3 ( 2.0) | 4.4 ( 2.4) | 0.3334 |
| % pain days | 52.7 (89.1) | 67.8 (75.3) | 0.1117 |
| % crisis days | 4.3 (14.2) | 5.8 (23.4) | 0.3119 |
| % days ED/hospital admits | 1.1 ( 4.1) | 0.0 ( 2.7) | 0.1478 |

Supplemental Table 2. Sample description comparing the gold-standard sample with the remainder of the N=232 PiSCES sample who submitted at least 30 diaries. Presented as frequency (%) of median (IQR: Interquartile range) for continuous variables.
